# Sure and Safe Method of Removing Foreign Bodies from the Ear

**Published:** 1872-10

**Authors:** 


Sure and Safe Method of Removing Foreign Bodies from the Ear.—The following method for removing foreign bodies from the external auditory meatus is suggested by Dr. Könenberg, of Paris (*Berliner Klinische Wochenschrift, No.* 9, iv 10, 1872), and is applicable in those cases in which the ordinary methods of extraction, by means of instruments and the syringe, have proved unavailing. It consists in smearing the presenting surface of the foreign body with some adhesive fluid, which speedily hardens, by the aid of which a sort of handle may be fastened to the body, which may then be readily withdrawn, without causing any pressure upon the walls of the auditory canal. One of the best substances to be employed for this purpose is ordinary *glue*, which should be dissolved in cold water the day before the operation, and then thickened by means of the water-bath immediately before being used. The glue is applied by means of a small brush, the head of the patient being allowed to rest upon a pillow. When the foreign body is well smeared, the end of the wooden brush-handle may be inserted into the auditory meatus, and placed in contact with the body. At the end of three-quarters of an hour, a firm union generally takes place, and the offending substance can be easily extracted. It is essential that the surface of the body upon which the glue is smeared should be perfectly dry. If the surface is made moist by the existence of some discharge, it may possibly be kept dry during the operation by the introduction of a current of warm air. If it is found impossible to keep it dry, then some other substance must be used to cause adhesion—such, for instance, as plaster of Paris or cement, which are hardened by the mixture of water. The writer is of the opinion that this so-called adhesive method can be applied in cases where the foreign body has penetrated the tympanum, provided the opening at the point of perforation is sufficiently large to admit of the introduction of a slender splinter of wood, to which the adhesive mixture has been applied, and by the aid of which the foreign substance may be laid hold of and extracted.—*Boston Medical and Surgical Journal.*

